# Dual Burden of Malnutrition Among Adolescents With Hunger Aged 12–15 Years in 41 Countries: Findings From the Global School-Based Student Health Survey

**DOI:** 10.3389/fmed.2021.771313

**Published:** 2022-01-10

**Authors:** Huaqing Liu, Min Zhang, Peipei Fu, Yan Chen, Chengchao Zhou

**Affiliations:** ^1^Centre for Health Management and Policy Research, School of Public Health, Cheeloo College of Medicine, Shandong University, Jinan, China; ^2^School of Public Health, Bengbu Medical College, Bengbu, China; ^3^School of Health Management, Bengbu Medical College, Bengbu, China; ^4^National Health Commission (NHC) Key Lab of Health Economics and Policy Research, Shandong University, Jinan, China; ^5^School of Public Health, Wannan Medical College, Wuhu, China

**Keywords:** hunger, obesity, adolescent, malnutrition, underweight, overweight

## Abstract

**Background:** Hunger is a pandemic among adolescents, resulting in both underweight and obesity, and posing a substantial health challenge.

**Objective:** To estimate the dual burden of malnutrition among adolescents with hunger.

**Design:** Data were from the Global school-based Student Health Survey (GSHS). In total, data from 26,986 adolescents with hunger across 5 regions and 41 countries between 2010 and 2015 were analyzed in this study. Weighted prevalence and mean estimates of underweight, overweight, and obesity were calculated by gender, age, and country. Prevalence and 95% confidence intervals (CI) were calculated for regional and country-level income.

**Results:** The total prevalence of underweight, overweight and obesity among young adolescents with hunger was 6.2% (95% CI: 4.4–8.0%), 25.1% (95% CI: 20.3–29.9%) and 8.9% (95% CI: 6.5–11.3%), respectively. Southeast Asia had the highest prevalence of underweight (17.2%; 95% CI: 7.3–27.0%). America had the highest regional prevalence of obesity (11.1%; 95% CI: 7.2–15.1%) and overweight (28.9%; 95% CI: 21.9–35.9%). Low income countries had relatively high prevalence of underweight (11.5%; 95% CI: 3.2–19.9%). High income countries had the highest prevalence of obesity (17.4%; 95% CI: 14.9–19.9%) and overweight (38.7%; 95% CI: 32.0–45.4%). The co-existence of underweight and overweight among adolescents with hunger was highest in the Eastern Mediterranean region, and in upper-middle and high-income countries.

**Conclusions:** There is a dual burden of underweight and obesity among adolescents with hunger aged 12–15 years, which differs between geographical regions. The integration of targeted interventions and policies is required to simultaneously address both underweight and increasing rates of obesity among adolescents with hunger in different regions.

## Introduction

The number of hungry people worldwide has been slowly rising since 2014, with more than 840 million people projected to experience hunger by 2030 (1); this issue is most acute among adolescents, with about 30% experiencing moderate or severe hunger, which is more than triple the global average for the total population of 8.9%. Hunger among adolescents is therefore a serious global public health issue. Adolescence is a critical period of rapid growth, in which the foundations for future health are established. Adolescents therefore have higher nutritional requirements, placing them at greater risk of malnutrition. Generally, hunger increases the risk of being underweight, which is a primary cause of poor health, and compromises the socio-economic development, ability to learn, and productivity of an individual (2).

Pediatric obesity is also increasing rapidly worldwide, with the global prevalence of obesity or overweight children and adolescents aged 5–19 years increasing more than four-fold from 4 to 18% from 1975 to 2016 (3). Overweight or obesity in adolescence is also likely to give rise to lifelong overweight or obesity (4), which is associated with an increased risk of developing chronic non-communicable diseases (such as type 2 diabetes, hypertension, and cardiovascular disease) at an earlier stage, as well as early death (5, 6).

Obesity is often a consequence of over-eating. A recent study indicated that hedonic hunger, defined as eating food for pleasure, is positively associated with obesity among women (7). Several studies have reported a positive association between hedonic hunger and eating unhealthy foods (8, 9) among young people, which increases the risk of childhood obesity (10). Among overweight or obese people, reducing hunger may improve weight loss (11).

Overweight or obesity and underweight may co-exist in a population (12, 13), referred to as the “dual burden” of hunger, which is a great challenge for public health that has negative economic impacts. This dual burden (14) was originally reported in adults, but is also known to affect young adolescents (2, 15); however, to date, there is a dearth of studies aiming to characterize the dual burden among hungry adolescents. Hunger may not only increase the risk of underweight, but may also be linked with obesity, posing complex and substantial challenges to adolescent health. In this study, we characterize the burden of underweight, overweight and obesity among young adolescents with hunger aged 12–15 years worldwide, using data from the World Health Organization (WHO) Global school-based Student Health Survey (GSHS). Describing the dual burden of hunger among adolescents will enable policymakers to identify and implement targeted actions to achieve the United Nations (UN) Sustainable Development Goal of ending malnutrition in all its forms by 2030.

## Materials and Methods

### Data and Participants

The data used in this study came from the GSHS, which is a global collaborative surveillance project designed to obtain and assess health behavioral risks and protective factors among young adolescents worldwide. It was developed by the World Health Organization in association with the United States Centers for Disease Control and Prevention, the United Nations Children Fund, the United Nations Educational, Scientific and Cultural Organization, and the Joint United Nations Programme on HIV/AIDS. The GSHS was approved in each country by a national government administration (most often the ministry of health or education) and an institutional review board or ethics committee. Verbal or written consent was obtained from participants and their parents in all countries. More details about the GSHS can be found at https://www.who.int/ncds/surveillance/gshs/en.

A two-stage cluster sampling strategy was used in each participating country. All schools were selected with a probability proportional to their enrolment sizes, then classes in which all students were eligible to participate in the survey were randomly chosen from these schools. The GSHS questionnaire was self-administered and anonymously completed by students. In total, 29,941 young adolescents with hunger aged 12–15 years from 5 regions and 41 countries completed the survey between 2010 and 2015. From these, 26,986 individuals were included in our study, after excluding participants with missing information on sex, age, height, weight, and hunger, and surveys in which response rates were <60%.

### Measures

Weight and height were self-reported in the GSHS, and body mass index (BMI) was calculated as weight in kilograms divided by the square of height in meters. Overweight, obesity and underweight were defined as BMI-for-age >1 standard deviation (SD), >2 SD, and <2 SD from the WHO growth reference values for 5–19 years (16, 17), respectively. Hunger was measured using the question “during the past 30 days, how often did you go hungry because there was not enough food in your home?” The possible responses were “never,” “rarely,” “sometimes,” “most of the time,” and “always.” In this study, respondents were categorized as hungry if the answer was “sometimes,” “most of the time,” or “always.” National incomes were stratified into four levels for analysis (“low income,” “lower-middle income,” “upper-middle income,” and “high income”) according to World Bank Analytical classifications based on gross national income per capita for the corresponding survey year (18).

### Statistical Analysis

All data were weighted using the cluster sampling design of the surveys, with stratification and primary sampling at the national level, to give nationally-representative samples. The weighted prevalence of underweight, overweight, and obesity were calculated for each country. The pooled prevalence and 95% confidence intervals (CI) for underweight, overweight, and obesity among all included individuals, boys, girls, those aged 12–13 years, and those aged 14–15 years, according to WHO region and national income, were calculated using Stata v16.0 (Stata Corporation, College Station, TX, USA). Differences with non-overlapping 95% CIs were considered statistically significant; which is a relatively conservative estimate. In addition, the chi-square test of independence was conducted for sex, age, WHO region, and national income against underweight, overweight, and obesity indices. A *P*-value of < 0.05 was considered statistically significant in this analysis.

## Results

### Participants

The characteristics of the individuals included in this study from the GSHS are described in Table 1. In total, 41 countries from five WHO regions had complete BMI and hunger data between 2010 and 2015 [Africa: 4; Western Pacific: 12; Southeast Asia: 2; Eastern Mediterranean: 10; and America (Central and South America): 13]. A total of 26,986 adolescents with hunger (46.7% of whom were male) aged 12–15 years were included in this study. The overall response rate was 90.1%, ranging from 70.5% (Timor Leste) to 97.5% (Brunei). The size of the samples varied from 87 (Chile) to 4,945 (Malaysia).

**Table 1:** Characteristics of the global school-based student health surveys by region and country (2010–2015).

	**Survey** **year**	**Response** **rate (%)**	**Sample** **size**	**Boys** **(%)**
**Africa (*****n*** **=** **4)**				
Algeria	2011	93.0	995	48.3
Mauritius and Rodriques	2011	92.3	493	45.2
Namibia	2013	92.6	873	42.0
Swaziland	2013	95.4	523	38.0
**Western pacific (*****n*** **=** **12)**				
Brunei	2014	97.5	553	43.8
Cambodia	2013	91.1	509	40.5
Cook Islands	2011	94.0	374	49.7
Fiji Islands	2010	91.9	778	43.2
Kiribati	2011	90.5	516	43.0
Malaysia	2012	95.7	4,945	50.6
Mongolia	2013	95.0	471	50.3
Nauru	2011	87.7	213	48.8
Philippines	2011	89.9	1,356	44.9
Solomon Islands	2011	81.9	569	50.3
Tonga	2010	95.5	1,030	43.7
Vietnam	2013	94.3	332	44.6
**Southeast asia (*****n*** **=** **2)**				
Bangladesh	2014	88.3	1,359	34.7
Timor leste	2015	70.5	409	35.9
**Eastern mediterranean (*****n*** **=** **10)**				
Afghanistan	2014	79.2	423	34.3
Egypt	2011	76.5	378	59.0
Iraq	2012	89.8	228	50.9
Kuwait	2015	85.9	468	42.1
Morocco	2010	91.9	491	51.5
Occupied palestinian territory	2010	83.9	2,225	52.4
Oman	2015	85.4	264	47.3
Sudan	2012	89.8	254	41.7
Syria	2010	91.1	855	43.0
United arab emirates	2010	87.5	287	39.7
**America (*****n*** **=** **13)**				
Bahamas	2013	89.4	353	44.5
Barbados	2011	84.2	294	49.3
Belize	2011	87.2	355	43.9
Bolivia	2012	92.9	706	52.5
Chile	2013	89.7	87	50.6
El salvador	2013	90.2	212	52.4
Guyana	2010	94.7	642	45.3
Honduras	2012	90.5	182	50.5
Jamaica	2010	94.9	517	50.7
Peru	2010	89.7	391	50.6
Saint kitts and nevis	2011	86.3	334	48.8
Trinidad and tobago	2011	82.5	564	54.1
Uruguay	2012	89.4	178	50.0

*Data are proportion (95% confidence interval)*.

### Prevalence of Underweight, Overweight and Obesity by Country

**Figure 1:**
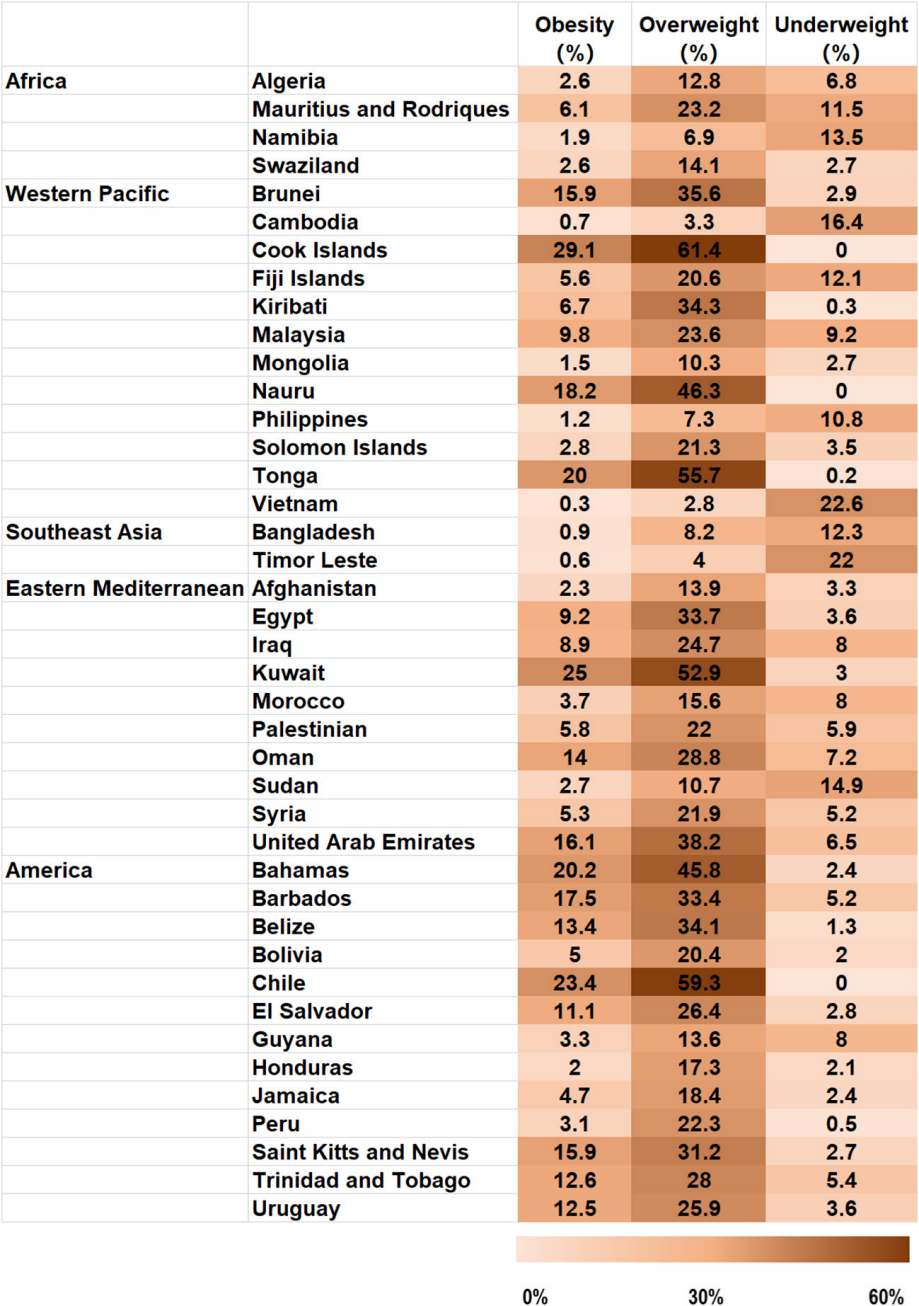
Proportions of obese, overweight and underweight adolescents with hunger aged 12–15 years, organized by country.

The prevalence of underweight, overweight, and obesity in the 41 countries included in our study is shown in Figure 1. There were considerable variations in the prevalence of underweight, overweight, and obesity across countries. Overall, 48.8% (20/41) countries had a prevalence of underweight ≥5.0%, with prevalences exceeding 15.0% in Vietnam, Timor Leste, and Cambodia. The prevalence of underweight was high in many countries in Southeast Asia. Conversely, the prevalence of underweight was <1.0% in the Cook Islands, Kiribati, Nauru, Tonga, Chile, and Peru. The prevalence of overweight varied from 2.8% in Vietnam to 61.4% in the Cook Islands. The prevalence of obesity was highest in Kuwait, Chile, Bahamas, Tonga, and Cook Islands (≥20%) and lowest in Cambodia, Vietnam, Bangladesh, and Timor Leste (<1.0%).

### Prevalence of Underweight, Overweight and Obesity by Region, Sex and Age

As shown in Table 2, the total prevalence of underweight, overweight and obesity among young adolescents across all 41 countries was 6.2% (95% CI: 4.4–8.0%), 25.1% (95% CI: 20.3–29.9%) and 8.9% (95% CI: 6.5% 95% CI: 11.3%), respectively. Southeast Asia had the highest prevalence of underweight, at 17.2% (95% CI: 7.3–27.0%), followed by Africa, at 8.6% (95% CI: 3.7–13.5%). America had the highest regional prevalence of obesity, at 11.1% (95% CI: 7.2–15.1%) and overweight, at 28.9% (95% CI: 21.9–35.9%). Sex, age and regional differences in the prevalence of obesity and underweight were calculated using the chi-square test. Regional differences were still apparent when the data were stratified by sex and age group; however, the total and regional prevalence values for obesity and underweight did not differ significantly between boys and girls, or between adolescents aged 12–13 and 14–15 years, as indicated by non-overlapping 95% CIs.

**Table 2:** Prevalence of obese, overweight and underweight adolescents with hunger by region, sex and age.

	**Obesity**	**Overweight**	**Underweight**
**All**	8.9% (6.5–11.3%)	25.1% (20.3–29.9%)	6.2% (4.4–8.0%)
**Gender**			
Boys	9.4% (6.8–12.1%)	24.3% (19.4–29.2%)	7.4% (5.4–9.4%)
Girls	8.3% (6.0–10.6%)	25.9% (20.9–30.9%)	5.1% (3.3–6.8%)
χ^2^	34.837	0.006	96.301
*P*	<0.001	0.937	<0.001
**Age group**			
12–13 years	10.4% (7.7–13.0%)	28.0% (23.1–32.8%)	5.9% (4.1–7.7%)
14–15 years	8.1% (5.7–10.5%)	23.8% (19.0–28.6%)	6.3% (4.4–8.2%)
χ^2^	68.277	91.809	19.240
*P*	<0.001	<0.001	<0.001
**All**			
Africa	3.3% (1.4–5.2%)	14.3% (7.5–21.1%)	8.6% (3.7–13.5%)
Western pacific	9.3% (3.8–14.8%)	26.9% (15.2–38.6%)	6.7% (2.4–11.1%)
Southeast Asia	0.8% (0.4–1.1%)	6.1% (1.9–10.3%)	17.2% (7.3–27.0%)
Eastern mediterranean	9.3% (4.7–13.9%)	26.2% (18.1–34.4%)	6.6% (4.3–8.8%)
America	11.1% (7.2–15.1%)	28.9% (21.9–35.9%)	3.0% (1.7–4.2%)
χ^2^	278.054	572.279	231.950
*P*	<0.001	<0.001	<0.001
**Boys**			
Africa	2.4% (−0.3–5.1%)	10.5% (5.2–15.8%)	12.1% (6.6–17.7%)
Western pacific	10.3% (4.4–16.3%)	25.5% (14.5–36.5%)	8.2% (3.1–13.3%)
Southeast Asia	0.5% (0.3–0.6%)	6.5% (1.5–11.4%)	17.5% (10.7–24.2%)
Eastern mediterranean	10.7% (6.0–15.8%)	27.6% (18.9–36.2%)	7.7% (5.6–9.8%)
America	11.2% (6.9–15.5%)	27.7% (19.7–35.6%)	3.4% (2.0–4.7%)
χ^2^	168.220	282.603	152.151
*P*	<0.001	<0.001	<0.001
**Girls**			
Africa	4.0% (2.6–5.4%)	17.5% (8.8–26.2%)	5.7% (1.5–10.0%)
Western pacific	8.3% (3.2–13.4%)	28.3% (15.7–41.0%)	5.3% (1.6–9.0%)
Southeast Asia	1.2% (0.2–2.2%)	5.6% (2.6–8.6%)	16.2% (2.7–29.6%)
Eastern mediterranean	7.9% (3.4–12.3%)	24.9% (16.9–32.9%)	5.5% (2.5–8.6%)
America	11.0% (6.9–15.1%)	30.2% (23.4–37.1%)	2.5% (1.3–3.8%)
χ^2^	126.151	320.705	117.875
*P*	<0.001	<0.001	<0.001
**12–13 years**			
Africa	5.1% (3.3–6.8%)	17.1% (10.4–23.7%)	8.5% (5.5–11.4%)
Western pacific	9.6% (4.1–15.2%)	28.3% (18.0–38.7%)	7.4% (3.0–11.8%)
Southeast Asia	2.1% (1.7–2.4%)	8.0% (6.4–9.6%)	17.3% (9.7–24.8%)
Eastern mediterranean	11.8% (5.5–18.2%)	31.1% (21.4–40.8%)	5.2% (3.4–7.0%)
America	12.9% (8.9–16.8%)	31.6% (23.8–39.5%)	2.6% (1.2–4.0%)
χ^2^	94.411	180.353	77.491
*P*	<0.001	<0.001	<0.001
**14–15 years**			
Africa	2.5% (0.5–4.5%)	12.9% (5.9–19.9%)	9.0% (3.1–14.8%)
Western pacific	9.2% (3.7–14.7%)	27.0% (15.1–39.0%)	6.5% (2.0–10.9%)
Southeast Asia	0.4% (0.2–0.6%)	5.6% (0.6–10.5%)	17.1% (6.4–27.7%)
Eastern mediterranean	7.8% (3.6–12.0%)	23.5% (15.8–31.1%)	7.1% (4.6–9.5%)
America	10.2% (6.0–14.4%)	27.3% (20.6–34.1%)	3.1% (1.9–4.3%)
χ^2^	188.054	390.197	154.872
*P*	<0.001	<0.001	<0.001

*Data are proportion (95% confidence interval)*.

### Prevalence of Underweight, Overweight and Obesity by Income, Sex and Age

Table 3 shows the prevalence of obesity, overweight, and underweight among adolescents with hunger by national income, sex, and age. Compared with high income countries (3.5%; 95% CI: 2.1–5.0%), low income countries had a significantly higher prevalence of underweight (11.5%; 95% CI: 3.2–19.9%). High income countries had the highest prevalence of obesity, at 17.4 % (95% CI: 14.9–19.9%) and overweight, at 38.7% (95% CI: 32.0–45.4%). This relationship was still observed in the data when stratified by sex and age group. We found significant differences in the prevalence of obesity, overweight, and underweight across different national income levels.

**Table 3:** Prevalence of obese, overweight and underweight adolescents with hunger by national income, sex and age.

**Income group**	**Obesity**	**Overweight**	**Underweight**
**All**			
Low income	1.9% (0.7–3.1%)	9.3% (2.9–15.7%)	11.5% (3.2–19.9%)
Lower middle income	5.3% (2.8–7.8%)	20.1% (13.8–26.4%)	6.8% (3.5–10.0%)
Upper middle income	5.3% (2.9–7.7%)	18.8% (13.7–23.9%)	7.4% (3.8–11.0%)
High income	17.4% (14.9–19.9%)	38.7% (32.0–45.4%)	3.5% (2.1–5.0%)
χ^2^	669.760	696.021	125.137
*P*	<0.001	<0.001	<0.001
**Boys**			
Low income	2.9% (0.8–5.0%)	10.2% (3.5–16.8%)	11.5% (2.7–20.3%)
Lower middle income	5.2% (2.8–7.5%)	18.3% (12.3–24.2%)	7.9% (4.3–11.4%)
Upper middle income	5.5% (2.0–8.9%)	17.1% (10.7–23.4%)	9.7% (4.9–14.6%)
High income	18.9% (16.1–21.7%)	39.9% (33.2–46.4%)	4.6% (2.7–6.4%)
χ^2^	311.322	329.579	66.835
*P*	<0.001	<0.001	<0.001
**Girls**			
Low income	0.7% (0.3–1.2%)	8.3% (1.3–15.2%)	11.8% (2.4–21.2%)
Lower middle income	5.3% (2.5–8.1%)	21.6% (14.7–28.5%)	5.7% (2.7–8.8%)
Upper middle income	5.2% (3.7–6.6%)	20.7% (15.5–25.9%)	5.2% (2.6–7.9%)
High income	16.1% (13.1–19.1%)	37.8% (30.3–45.3%)	2.6% (1.2–4.1%)
χ^2^	361.998	370.617	68.989
*P*	<0.001	<0.001	<0.001
**12–13 years**			
Low income	1.8% (−1.0–4.5%)	11.4% (4.4–18.8%)	8.6% (2.4–14.7%)
Lower middle income	6.3% (3.7–8.9%)	22.6% (16.9–28.4%)	7.1% (3.7–10.5%)
Upper middle income	7.5% (4.4–10.6%)	21.6% (15.2–28.0%)	6.9% (3.2–10.5%)
High income	20.0% (16.7–23.3%)	42.8% (35.3–50.2%)	3.3% (1.8–4.7%)
χ^2^	223.694	206.968	45.597
*P*	<0.001	<0.001	<0.001
**14–15 years**			
Low income	1.8% (0.2–3.4%)	8.7% (2.6–14.8%)	12.2% (3.1–21.3%)
Lower middle income	4.7% (2.3–7.1%)	19.0% (12.7–25.3%)	6.8% (3.5–10.1%)
Upper middle income	4.1% (2.0–6.2%)	17.1% (12.3–21.9%)	7.5% (3.6–11.4%)
High income	16.1% (12.9–19.3%)	36.9% (30.2–43.5%)	3.6% (2.1–5.2%)
χ^2^	433.345	325.758	79.963
*P*	<0.001	<0.001	<0.001

*Data are proportion (95% confidence interval)*.

### Relationship Between Prevalence of Overweight and Prevalence of Underweight

**Figure 2:**
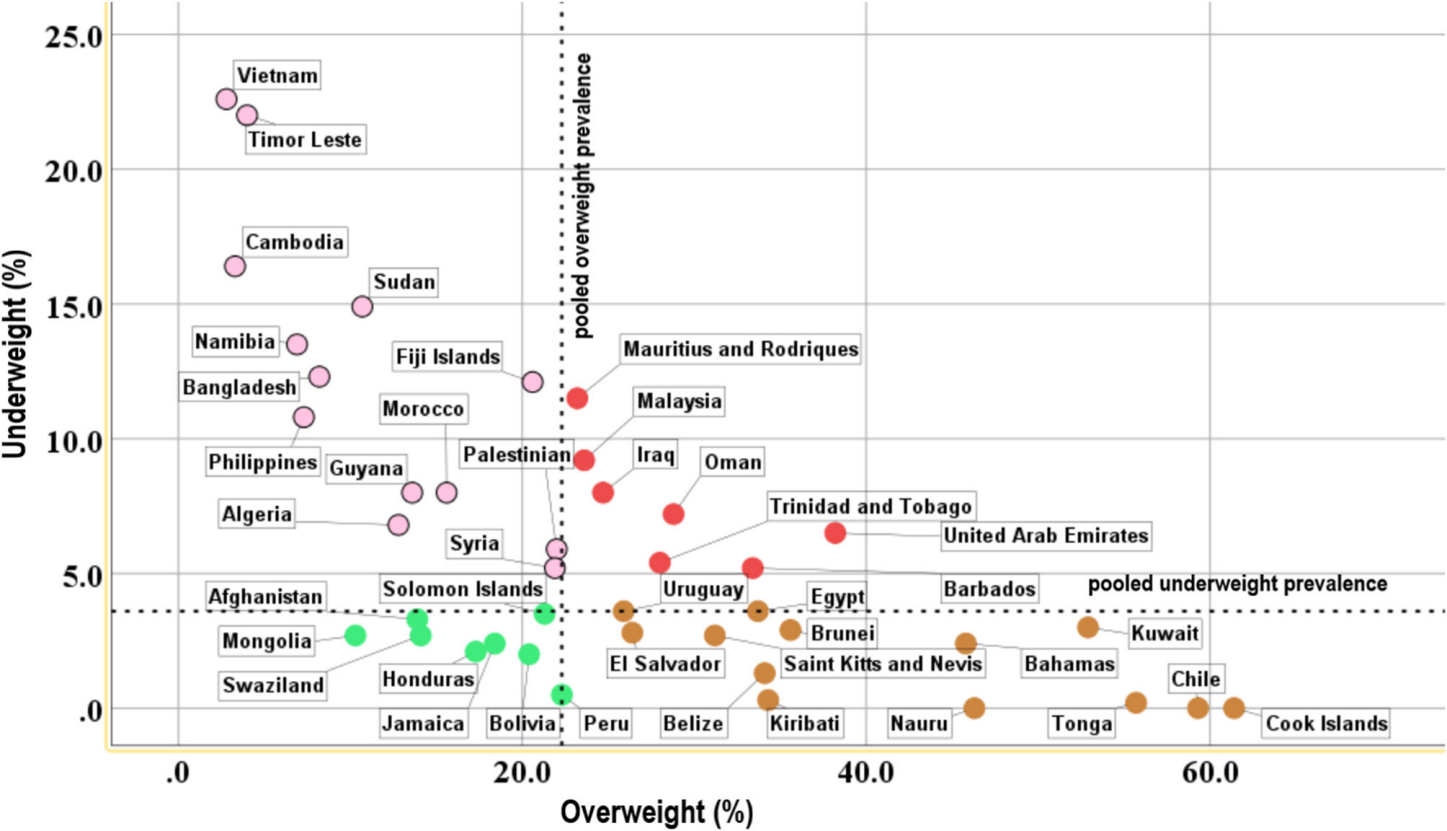
Scatterplot showing the relationship between the proportion of overweight and underweight adolescents with hunger aged 12–15 years, organized by country.

Figure 2 shows the relationship between prevalence of overweight and that of underweight by among adolescents with hunger by country. 17.1% (7/41) countries had a prevalence of overweight and underweight greater than the median prevalence estimates for overweight and underweight, respectively, with the highest values observed in the Eastern Mediterranean region (30.0%; 3/10), and in the upper-middle and high-income countries (38.9%; 7/18).

## Discussion

In this study, we estimated the dual burden of malnutrition among adolescents with hunger at the population level from 41 countries around the world. We found that 1 in 15 adolescents with hunger was underweight, and 1 in 4 were overweight or obese. Adolescent populations with hunger contained those who were underweight and those who were overweight or obese.

Our findings showed a high prevalence of underweight, overweight, and obesity among young adolescents with hunger; however, there were also substantial differences between regions and countries. The prevalence of underweight was higher in Southeast Asia, while the prevalence of obesity was higher in Central and South America, which is comparable with findings from other studies (3, 19). More adolescents with hunger were underweight than obese in the low and lower-middle income countries, as well as all countries across Southeast Asia and Africa. However, pediatric obesity has surpassed underweight in Central and South America, in which most countries are high income. Obesity is often associated with high income countries or high socio-economic status (20, 21). The co-existence of underweight and overweight among adolescents with hunger at the national level was highest among Eastern Mediterranean countries and those with upper-middle and high incomes. Despite the increasing prevalence of overweight and obesity worldwide, our finding that young adolescents with hunger who were underweight outnumbered those with obesity in most countries indicated that underweight remains a primary health issue in young adolescents with hunger; there is thus a continued need for policies that enhance food security, especially in Southeast Asia, Africa, and low-income countries.

The prevalence of adolescents with hunger who were underweight in this study (7.6%) was greater than that of young adolescents in a previous study by Yang and colleagues (4.7%) (19). Adolescents with hunger due to food insufficiency at home were more likely to be underweight when faced with additional food shortages or food poverty. However, adolescents with hunger who are exposed to obesogenic environments (available and affordable food outside the home, especially foods that are high in fat or sugar, low in vitamins, minerals and protein, and relatively inexpensive) are more likely to become overweight or obese (22). Previous research has shown that greater degrees of hunger lead to the consumption of greater amounts of food, owing to increasing meal size or frequency (23). Furthermore, hunger may cause food deprivation, which leads to stress, anxiety, and emotional distress (24, 25), which may result in stress eating or over-eating (26), and visceral fat accumulation (27). Thus, adolescents with hunger may actually be more likely to become obese than those without hunger (12, 28). Levels of overweight among adolescents with hunger in this study were comparable with those reported in other studies (29).

It is remarkable that middle-income countries may have the greatest dual burden of malnutrition. One recent study (30) showed that the prevalence of obesity is highest among poor individuals in high income countries, and among wealthy individuals in low income countries. The relatively rapid transition from underweight to overweight and obesity has been noted in low and middle income countries (31, 32). Increasing economic development may lead to greater availability of nutrient-poor, energy-dense foods and thus a transition from underweight to overweight and obese among young adolescents; strategies targeting this phenomenon may therefore help to reduce levels of malnutrition in developing countries. Notably, the transition from underweight to overweight or obese among adolescents has accelerated in Southeast Asia (15), indicating that a strategy to combat malnutrition in this region should focus on attenuating this transition while simultaneously resolving hunger issues leading individuals to become or stay underweight.

The dual burden of malnutrition poses serious resource allocation challenges for governments in order to address the dual burden of underweight and overweight or obesity simultaneously (33, 34). Policies that target overweight and obesity in adolescents involve using taxes on “unhealthy” foods high in fat, sugar and/or salt to reduce their consumption, particularly in high income countries. Evidence from Central America indicates that tariff removal has had a positive effect on the affordability of healthy balanced diets and helped improve nutrition in a region characterized by the coexistence of underweight and obesity (35). Price subsidies or food vouchers that target whole grains and fresh fruits and vegetables could also be adopted to make nutritionally dense food more affordable. In addition, supply-side reforms in the food industry such as tax cuts could help to stimulate the production and supply of healthier food. The unaffordability of “healthy” food leads to economic inequalities in levels of overweight and obesity, and also limits the impact of policies targeting unhealthy foods.

To our knowledge, this study is the first to assess the dual burden of malnutrition in adolescents with hunger from a global perspective. However, there are some limitations to our study. First, weight and height were self-reported, which may lead to underestimation of the actual number of overweight or obese individuals (36–38). Second, the GSHS has a wide time frame of data collection (from 2010 to 2015) limiting the comparability of data across countries or regions.

## Conclusions

The nutritional landscape among adolescents with hunger is becoming more complex. We found a dual burden of malnutrition (underweight and overweight or obese) among adolescents with hunger aged 12–15 years, which varied by geographical region. Our findings highlighted that regionally-targeted, integrative interventions and policies are needed to simultaneously address underweight and increasing levels of obesity among adolescents with hunger.

## Data Availability Statement

The original contributions presented in the study are included in the article/supplementary material, further inquiries can be directed to the corresponding author/s.

## Ethics Statement

The studies involving human participants were reviewed and approved by the GSHS was approved, in each country, by both a national government administration (most often the ministry of health or education) and an institutional review board or ethics committee. Verbal or written consent was obtained from participants and their parents in all countries. Written informed consent to participate in this study was provided by the participants' legal guardian/next of kin. Written informed consent was obtained from the individual(s), and minor(s)' legal guardian/next of kin, for the publication of any potentially identifiable images or data included in this article.

## Author Contributions

HL conceptualized and designed the study, drafted the initial manuscript, and revised the manuscript. MZ, PF, and YC collated the data, carried out the initial analyses, and reviewed and revised the manuscript. CZ conceptualized and designed the study and critically reviewed the manuscript. All authors approved the final manuscript as submitted and agree to be accountable for all aspects of the work.

## Funding

This study was supported by the National Natural Science Foundation of China (71974117), the Natural Science Research Project of Anhui Educational Committee (KJ2019A0302), and the 512 Talent training Project of Bengbu Medical College (BY51201203). The funding bodies had no role in designing this study, collecting, analyzing, or interpreting the data, producing the draft manuscript, or deciding to publish the results of this study.

## Conflict of Interest

The authors declare that the research was conducted in the absence of any commercial or financial relationships that could be construed as a potential conflict of interest.

## Publisher's Note

All claims expressed in this article are solely those of the authors and do not necessarily represent those of their affiliated organizations, or those of the publisher, the editors and the reviewers. Any product that may be evaluated in this article, or claim that may be made by its manufacturer, is not guaranteed or endorsed by the publisher.
